# Structural Basis for Dimeric Genome Selection by HIV-1

**DOI:** 10.64898/2026.06.17.732839

**Published:** 2026-10-22

**Authors:** Nele Merret Hollmann, Siarhei Kharytonchyk, Angela Buchannan, Caela Phillips, Amir Ledbetter, Fairine Ahmed, Anna Adelstein, Kaylee Barry, Samuel A. Geleta, Cleo Burnett, Alice Telesnitsky, Barbie K. Ganser-Pornillos, Owen Pornillos, Michael F. Summers

## Abstract

HIV-1 selects and packages a dimeric RNA genome into assembling virions, a requirement for strand transfer-mediated recombination during reverse transcription. Conserved RNA elements within the 5’-leader that promote dimerization and packaging have been identified (dimer initiation signal, DIS; core encapsidation signal, Ψ ^CES^ ; respectively), but the molecular mechanism that couples these processes remains unknown. Using an in vitro virus assembly assay, we show that dimeric HIV-1 5’-Leader RNAs promote assembly of viral Gag proteins into protein lattices resembling those of immature virions, whereas monomeric 5’-Leader constructs with similar affinities for Gag do not. Assembly requires the cellular factor, inositol hexakisphosphate, mimicking biology. Cryogenic electron microscopy (cryoEM) and tomography (cryoTM) studies of nascent assemblies are consistent with a structural model in which the dimeric DIS _2_ helix bridges two elongated 6-helix bundles formed by the SP1 domains of adjacent Gag hexamers. The structure positions clusters of RNA-binding zinc knuckles from Gag’s nucleocapsid domain in close proximity to clusters of conserved guanosines shown here to be essential for genome packaging. Our findings suggest that DIS _2_ functions as a molecular ruler, organizing Gag hexamers into structures optimized for Gag recruitment and Gag lattice expansion.

